# Anti-Inflammatory Activity of a Novel Family of Aryl Ureas Compounds in an Endotoxin-Induced Airway Epithelial Cell Injury Model

**DOI:** 10.1371/journal.pone.0048468

**Published:** 2012-11-08

**Authors:** Nuria E. Cabrera-Benitez, Eduardo Pérez-Roth, Milena Casula, Ángela Ramos-Nuez, Carla Ríos-Luci, Carlos Rodríguez-Gallego, Ithaisa Sologuren, Virginija Jakubkiene, Arthur S. Slutsky, José M. Padrón, Jesús Villar

**Affiliations:** 1 CIBER de Enfermedades Respiratorias, Instituto de Salud Carlos III, Madrid, Spain; 2 Multidisciplinary Organ Dysfunction Evaluation Research Network, Research Unit, Hospital Universitario Dr. Negrin, Las Palmas de Gran Canaria, Spain; 3 BioLab, Instituto Universitario de Bio-Orgánica “Antonio González” (IUBO-AG), Universidad de La Laguna, La Laguna, Spain; 4 Department of Immunology, Hospital Universitario Dr. Negrin, Las Palmas de Gran Canaria, Spain; 5 Department of Organic Chemistry, Faculty of Chemistry, Vilnius University, Vilnius, Lithuania; 6 Keenan Research Center at the Li Ka Shing Knowledge Institute of St. Michael's Hospital, Toronto, Canada; 7 Interdepartmental Division of Critical Care Medicine, University of Toronto, Toronto, Canada; University of Giessen Lung Center, Germany

## Abstract

**Background:**

Despite our increased understanding of the mechanisms involved in acute lung injury (ALI) and the acute respiratory distress syndrome (ARDS), there is no specific pharmacological treatment of proven benefit. We used a novel screening methodology to examine potential anti-inflammatory effects of a small structure-focused library of synthetic carbamate and urea derivatives in a well established cell model of lipopolysaccharide (LPS)-induced ALI/ARDS.

**Methodology/Principal Findings:**

After a pilot study to develop an *in vitro* LPS-induced airway epithelial cell injury model, a library of synthetic carbamate and urea derivates was screened against representative panels of human solid tumor cell lines and bacterial and fungal strains. Molecules that were non-cytotoxic and were inactive in terms of antiproliferative and antimicrobial activities were selected to study the effects on LPS-induced inflammatory response in an *in vitro* cell culture model using A549 human alveolar and BEAS-2B human bronchial cells. These cells were exposed for 18 h to LPS obtained from *Escherichia coli*, either alone or in combination with the test compounds. The LPS antagonists rhein and emodin were used as reference compounds. The most active compound (CKT0103) was selected as the lead compound and the impact of CKT0103 on pro-inflammatory IL-6 and IL-8 cytokine levels, expression of toll-like receptor-4 (TLR4) and nuclear factor kappa B inhibitor alpha (IκBα) was measured. CKT0103 significantly inhibited the synthesis and release of IL-6 and IL-8 induced by LPS. This suppression was associated with inhibition of TLR4 up-regulation and IκBα down-regulation. Immunocytochemical staining for TLR4 and IκBα supported these findings.

**Conclusions/Significance:**

Using a novel screening methodology, we identified a compound – CKT0103 – with potent anti-inflammatory effects. These findings suggest that CKT0103 is a potential target for the treatment of the acute phase of sepsis and sepsis-induced ALI/ARDS.

## Introduction

Acute lung injury (ALI) and its more severe form, the acute respiratory distress syndrome (ARDS), is a relatively common syndrome in critically ill patients associated with high morbidity and mortality [1]. Data from the ALIVE Study [2] showed that about 7% of all intensive care unit patients develop ALI/ARDS, and that sepsis is the most common predisposing factor. Sepsis is characterized by a systemic inflammatory state in response to circulating microbes or microbial toxins such as lipopolysaccharide (LPS) or bacterial DNA [3]. The ultimate cause of death in septic patients is usually development of multiple system organ failure, which frequently starts as lung dysfunction [1], [4].

LPS, also termed endotoxin, is the major component of the outer membrane of gram-negative bacteria and is a common trigger of sepsis [5]. Toll-like receptors (TLRs), which function as sensors of microbial infection, recognize LPS and are critical for the initiation of inflammatory and immune defense responses [6]. A major downstream effect of TLR signaling is activation of the transcription nuclear factor kappa B (NF-κB), which eventually leads to expression of many genes related to innate immunity and inflammation, and other gene products [6]–[8].

Despite intense research and an increased understanding of the pathophysiological processes involved, there are no specific pharmacological treatments of proven benefit for ALI/ARDS [9], [10]. One approach for identifying novel therapies for various diseases is to use large scale screening of various molecules with appropriate simple models. Using a screening strategy based on a well established protocol of the National Cancer Institute (NCI) of the United States [11], we have previously identified targets that are active in various forms of cancer [12], [13]. We wondered whether a similar screening program would be able to identify novel candidate molecules which might be effective in attenuating or inhibiting the inflammatory response which ultimately lead to LPS/sepsis-induced ALI/ARDS.

Using an *in vitro* LPS-induced pulmonary epithelial injury model based on the first steps in the development of sepsis/ALI [14]–[20], we screened a library of about 300 small molecules possessing a wide diversity of chemical structures, and identified a group of novel pyrimidinyl carbamates and pyrimidinyl ureas as potential therapeutic candidates in sepsis/ALI. These carbamates and ureas can be synthesized from the corresponding (pyrimidin-4-yloxy)- and (pyrimidin-3-yl)-acetohydrazides, as reported previously [21]. The most active compound (CKT0103) was selected as the lead compound and further used to investigate the capacity of pharmacological inhibition of TLR4 signaling in LPS-stimulated human A549 alveolar and BEAS-2B bronchial pulmonary epithelial cells.

## Materials and Methods

### Reagents

All chemicals used in this study were commercially available and research-grade. File S1 for reagent details. To the best of our knowledge, there is no standard drug to be used as a reference in the treatment of LPS-induced effects. Hence, we selected rhein and emodin as reference compounds for our experimental study since both compounds are commercially available natural products from traditional herbs that have been shown to inhibit LPS-induced NF-κB activation and inflammatory cytokine expression [22]. A subset from our library of 2,000 compounds was selected for this study; the only requisite was that the compounds to be studied were non-cytotoxic and non-antimicrobial (see ESM, for details).

### LPS-induced airway epithelial cell injury models

We chose A549 cells [human pulmonary alveolar epithelial carcinoma cells (ATCC, Manassas, VA, USA)] and BEAS-2B cells [human bronchial epithelial cells (ATCC, Manassas, VA, USA)], as representative airway epithelial cell lines [14] to study the effects of the synthetic derivatives on the ability to inhibit LPS-induced effects in the airway epithelium These cells have been extensively used to study LPS-induced activation of pro-inflammtory cytokines, as an *in vitro* model based on the first steps in the development of sepsis-induced ALI/ARDS [14]–[20], [23]. A549 and BEAS-2B cells were cultured as previously described, maintained in 75 cm^2^ flasks in DMEM and DMEN/F-12, respectively, supplemented with 10% FBS, in a 37°C, 5% CO_2_, 95% humidified air incubator.

Exponentially growing A549 and BEAS-2B cells were trypsinized and re-suspended in 2% FBS antibiotic containing medium (100 units penicillin G and 0.1 mg of streptomycin per mL). Single cell suspensions displaying >97% viability, by using the trypan blue dye exclusion method according to the standard protocol [24], were subsequently counted. After counting, dilutions were made to give the appropriate cell densities for inoculation onto 96-well microtiter plates. Cells were inoculated in a volume of 100 µL per well at a density of 3×10^4^ cells per well. After 24 h, cells were exposed to *Escherichia coli* (*E. coli*) LPS (0.1, 1.0, 10, 100 ng/mL) for 6, 12, and 18 hours. LPS was obtained from *E. coli* serotype 055:B5 (Sigma-Aldrich, St Louis, MO, USA). In our preliminary studies, A549 and BEAS-2B cell survival decreased with increasing concentration of *E. coli* LPS. The lowest survival was observed with 100 ng/mL of LPS during 18 hours. This LPS concentration and time-period were used for the subsequent experiments (see ESM and Figure S1 for details).

### Screening methodology to test the inhibition of LPS effects

A549 and BEAS-2B cells were grown in 96-well microtiter plates in a volume of 100 µL per well at a density of 3×10^4^ cells per well. After 24 h, cells were exposed to 100 ng/mL LPS for 18 hours either alone or in combination with each test or reference drug at a final concentration of 10 µM. This concentration was chosen based on preliminary antiproliferative assays. We tested 11 synthetic compounds (see ESM for details). Since the best results in terms of cell survival were observed for the derivative 2e (CKT0103), this compound was selected for further testing in our cell lines (see ESM and Table S1 for details).

Additionally, we tested: (i) LPS (100 ng/mL) either alone or in combination with 0.1, 1, 10, 100, and 1000 µM CKT0103, and (ii) LPS (100 ng/mL) either alone or in combination with 0, 5, 10, 20 µM CKT0103. Tested compound was non-toxic to cells at a dose of 100 ng/mL *E. coli* LPS and 10 µM CKT0103. For all experimental conditions and after incubation for 18 h, cells were precipitated with 25 µL ice-cold TCA (50% w/v) and fixed for 60 min at 4°C. Then, the sulforhodamine B (SRB) assay [25] was performed. The optical density (OD) of each well was measured at 492 nm, using BioTek's PowerWave XS Absorbance Microplate Reader. The percentage of surviving cells (PS) was calculated for each dose as: PS = 100×[(*T_D_*-*T_LPS_*)/(*C*-*T_LPS_*)], where *T_D_* represents the OD of wells treated with the drug at drug dose D, *C* stands for the OD of untreated cell wells (negative control-vehicle), and *T_LPS_* corresponds to the OD of wells of cells treated only with LPS (positive control). With this calculation, a PS value of 0 corresponds to the effect of treating cells with LPS alone, while positive PS values denote net cell survival. Negative PS values represent either cytotoxicity of the tested compound, or a synergistic interaction with LPS.

### Assessment of A549 and BEAS-2B cell viability

The viability of A549 and BEAS-2B cells was measured performing a dose-response curve of 0.1, 1, 10, 100, 1000 µM CKT0103 with 100 ng/mL *E. coli* LPS using the trypan blue exclusion method [24] (see ESM and Figure S2 for details).

### Analysis of changes in cell morphology

A549 and BEAS-2B cells were suspended at 5×10^6^ cells/flask and inoculated in 75 cm^2^ flasks for 24 h. Cells were then exposed to LPS (100 ng/mL) either alone or in combination with 10 µM rhein, emodin or the lead compound CKT0103 (Table S1). Then, cells were examined and photographed (Olympus Camedia digital camera, ×400 objective) under a phase-contrast microscope (Olympus CK-40 F-200) at the end of compound exposure.

### Measurement of Cytokines levels

We measured IL-6 and IL-8 cytokine levels in the cell culture media collected from A549 and BEAS-2 cells by enzyme-linked immunosorbent assay (ELISA) following standard techniques. After 18 h of drug incubation, media from each well were collected and stored at −80°C. Quantitative human IL-6 and IL-8 levels were measured using a flow cytometry-based bead array system in a FACS Calibur flow cytometer, and analyzed with CellQuest Pro software according to the manufacturer's protocols (BD Biosciences. San Jose, CA, USA). The results were expressed as picograms of released cytokine/10^6^ adherent cells (pg/10^6^ cells). The theoretical limit of detection for each protein using the BD CBA Human Inflammatory Cytokines Kit is defined as the corresponding concentration at two standard deviations above the median fluorescence of 20 replicates of the negative control (0 pg/mL).

### Protein Extraction and Immunoblotting

After 18 h of compound incubation, A549 and BEAS-2B cells from each experimental group were harvested and centrifuged at 1200 rpm for 7 minutes. Cell pellets were re-suspended in a cold lysis buffer (1% Nonidet P-40, 25 mM Tris-HCl (pH 7.5), 150 mM sodium chloride, 1 mM EDTA, 5 mM sodium fluoride, 1 mM sodium orthovanadate, 1 mM phenylmethylsulfonyl fluoride) plus Protease Inhibitor Cocktail and incubated for 10 min on ice. The supernatant, containing predominantly total proteins, was collected after 5 min centrifugation at 14,000 rpm at 4°C. Protein concentrations were determined with the Bio-Rad DC protein assay.

For immunoblotting, each sample was reduced in SDS-PAGE loading buffer prior to being boiled and then separated by SDS-PAGE (10%) and visualized by colloidal Coomassie staining. After this, samples were electroblotted onto polyvinylidene difluoride (PVDF) membrane, blocked for 1 h at room temperature in 5% non-fat milk, and probed with anti-TLR4 and anti-IκBα antibodies (Santa Cruz Biotechnology, Santa Cruz, CA). Following incubation with the corresponding peroxidase-conjugated secondary antibody (Goat Anti-rabbit IgG-HRP; Santa Cruz Biotechnology, Santa Cruz, CA), immunoblots were stripped using Restore Western Blot Stripping Buffer for 15 min at room temperature and re-probed with an anti-β-actin (Cell Signaling Technology, MA) antibody. Chemiluminiscence detection was performed with the ECL kit. Densitometry was performed using Scion Image software package. Western blots were repeated three times in each experimental condition.

### Immunocytochemistry for TLR4 and IκBα

Immunocytochemical stains were performed by applying a standard avidin-biotin complex (ABC) technique (see ESM for details). We used primary antibodies directed against TLR4 and IκBα (Santa Cruz Biotechnology Inc, Santa Cruz, CA). Staining was visualized using the 3-amino-9-ethylcarbazole AEC+/substrate Chromogen. Images were viewed using an Olympus (BX50) microscope and photographed with an Olympus Camedia digital camera at ×400 magnifications.

### Statistical Analysis

Data were expressed as mean ± standard deviation (SD) and analyzed using Graph Pad Prism version 5.0 software. Comparisons involving all experimental groups were performed with one-way analysis of variance (ANOVA). We used a Bonferroni correction for multiple comparisons. For Western blot experiments, the effects on TLR4 and IκBα were analyzed by the same statistical analyses using densitometric data normalized to β-actin as loading controls. Data are from three independent experiments. Data analyses were performed using SPSS (version 15.0 for Windows). Effects were considered to be statistically significant when p<0.05.

## Results

### Inhibition of LPS-induced effects

**Figure 1:**
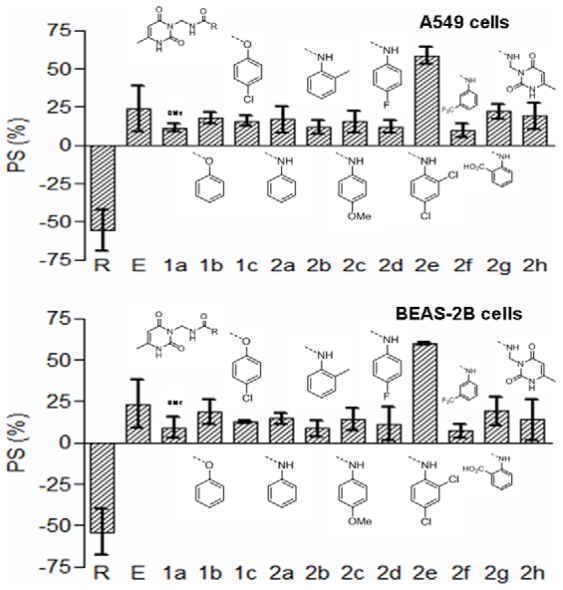
Percent of survival (PS) of 100 ng/mL LPS-stimulated A549 and BEAS-2B cells in combination with 10 µM rhein (R), emodin (E), and the rest of synthetic products. **1a–c:** pyrimidinyl carbamates; **2a–h:** pyrimidinyl ureas for 18 hours. The chemical structures of the products under evaluation are shown.

**Figure 2:**
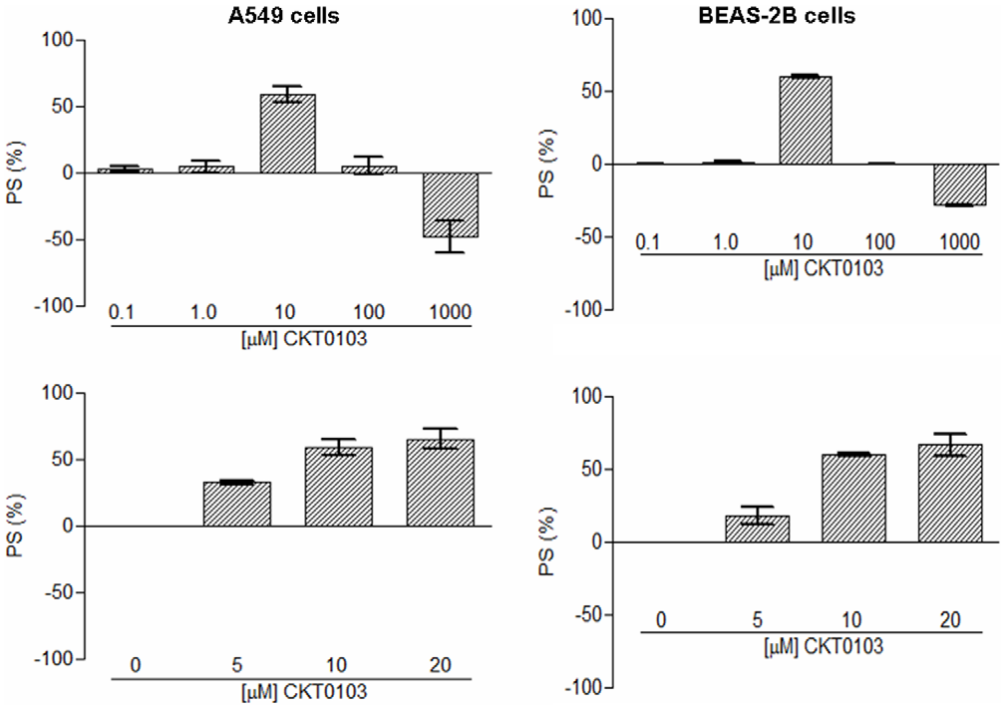
Percent of survival (PS) of 100 ng/mL LPS-stimulated A549 and BEAS-2B cells in combination with 0.1-1-10-100-1000 µM CKT0103 or in combination with 0-5-10-20 µM CKT0103 for 18 hours.

Tested synthetic compounds diminished the effects induced by LPS. The percentages of cell survival (PS) values were in the range 9.42 to 58.72% in A549 cells and 7.25 to 59.96% in BEAS-2B cells (Figure 1, Table S1). In general, they represented an improvement to the reference compounds rhein and emodin. The best results were obtained for derivative 2e (CKT0103) (N-(2,4-dichlorophenyl)-N′-[(6-methyl-2,4-dioxo-1,2-dihydropyrimidin-3(4H)-yl) methyl]urea), with a PS value of 58.72% in A549 cells and 59.96% in BEAS-2B cells. With the exception of this compound, there were only small differences in the biological activity between carbamate (1a–c) and urea (2a–h) derivatives, and between electron donor and electron withdrawing substituents on the aromatic ring. Based on this result, CKT0103 was selected for further testing. CKT0103 (at a concentration of 10 µM) markedly inhibited LPS-induced effects in A549 and BEAS-2B cells (Figure 2).

### CKT0103 does not reduce A549 and BEAS-2B cell viability

CKT0103 only had a significant effect on A549 and BEAS-2B cell viability at a concentration of 1000 µM (Figure S2).

### Morphological changes

**Figure 3:**
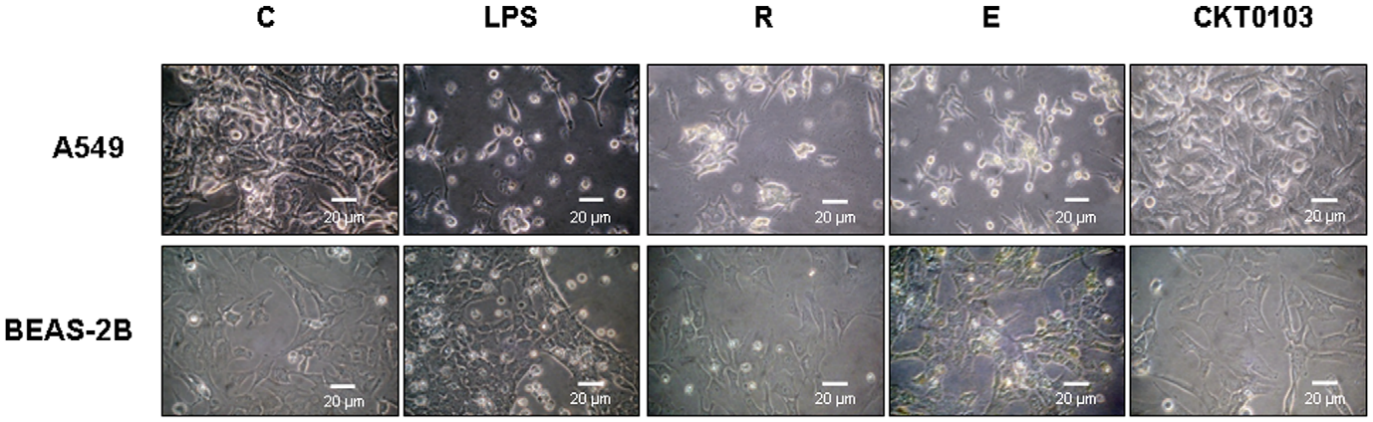
Morphological changes in A549 and BEAS-2B cells after treatment with 100 ng/mL *E. coli* LPS either alone or in combination with 10 µM rhein (R), emodin (E), CKT0103. We used A549 and BEAS-2B cells as control-vehicle (**C**) incubated in the presence of vehicle-0.5% (v/v) DMSO. ×400 magnifications.

Control-vehicle A549 and BEAS-2B cells incubated in the presence of vehicle-0.5% (v/v) DMSO grew as a monolayer with individual cells having typical epithelial shape. After 18 h of 100 ng/mL *E. coli* LPS stimulation, A549 and BEAS-2B cells became less confluent, more rounded, and detached from the well. These changes were also observed in cells exposed to *E. coli* LPS plus rhein and emodin. When cells were treated with 100 ng/mL *E. coli* LPS plus 10 µM CKT0103, cell detachment was prevented and a higher number of cell-cell contacts were observed (Figure 3).

### Pro-inflammatory cytokine levels

**Figure 4:**
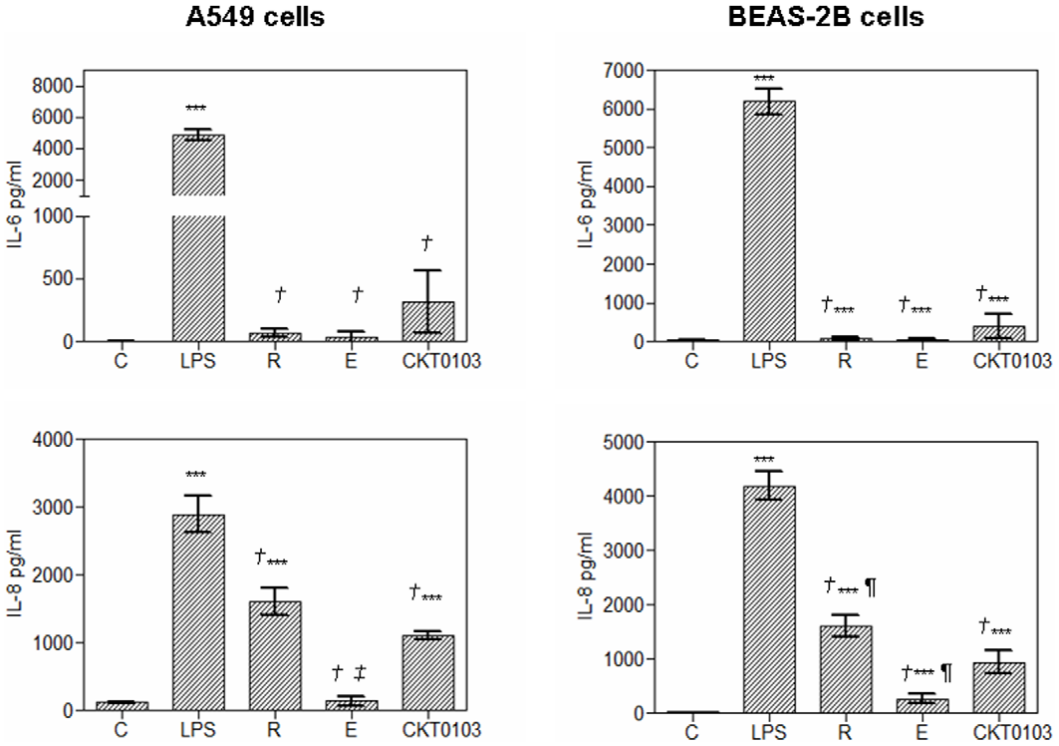
Pro-inflammatory IL-6 and IL-8 production by A549 and BEAS-2B cells as determined by CBA and flow cytometry analysis of culture supernatant after 18 hours of 100 ng/mL *E. coli* LPS stimulation either alone or in combination with 10 µM rhein, emodin and CKT0103. ***p<0.001 vs. control-vehicle (C); *†* p<0.001 vs. LPS; *¶* p<0.05 vs. LPS+ CKT0103; *‡* p<0.001 vs. LPS+CKT0103.

The reference compounds and CKT0103 reduced IL-6 and IL-8 levels induced by LPS alone in A549 and BEAS-2B cells (p<0.001, Figure 4).

### TLR4 and IκBα protein levels

**Figure 5:**
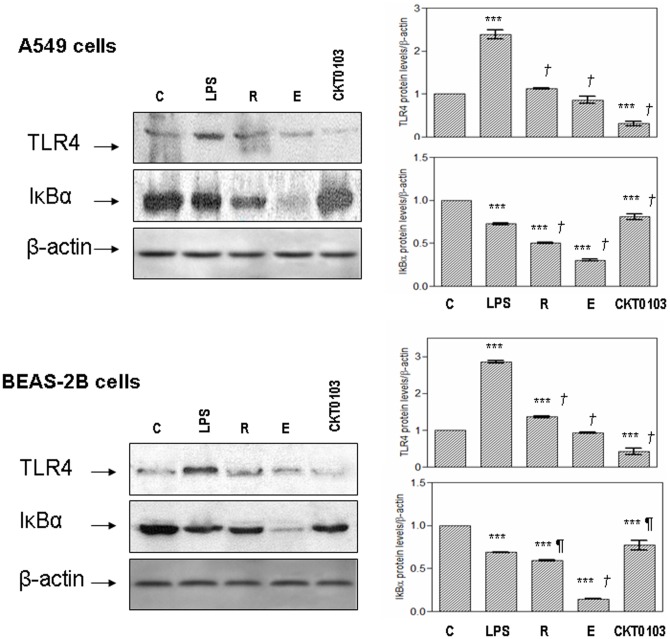
Immunobloting analysis for TLR4 and IκBα in A549 and BEAS-2B cells after stimulation with 100 ng/mL *E. coli* LPS either alone or in combination with 10 µM rhein, emodin and CKT0103 for 18 hours. **C:** Control-vehicle group; **LPS:** A549 and BEAS-2B cells stimulated with 100 ng/mL *E. coli* LPS; **R:** A549 and BEAS-2B cells stimulated with 100 ng/mL *E. coli* LPS in combination with 10 µM rhein for 18 hours; **E:** A549 and BEAS-2B cells stimulated with 100 ng/mL *E. coli* LPS in combination with 10 µM emodin for 18 hours; **CKT0103:** A549 and BEAS-2B cells stimulated with 100 ng/mL *E. coli* LPS in combination with 10 µM CKT0103 for 18 hours. ***p<0.001 vs. control-vehicle (C); *¶* p<0.05 vs. LPS; *†* p<0.001 vs. LPS.

**Figure 6:**
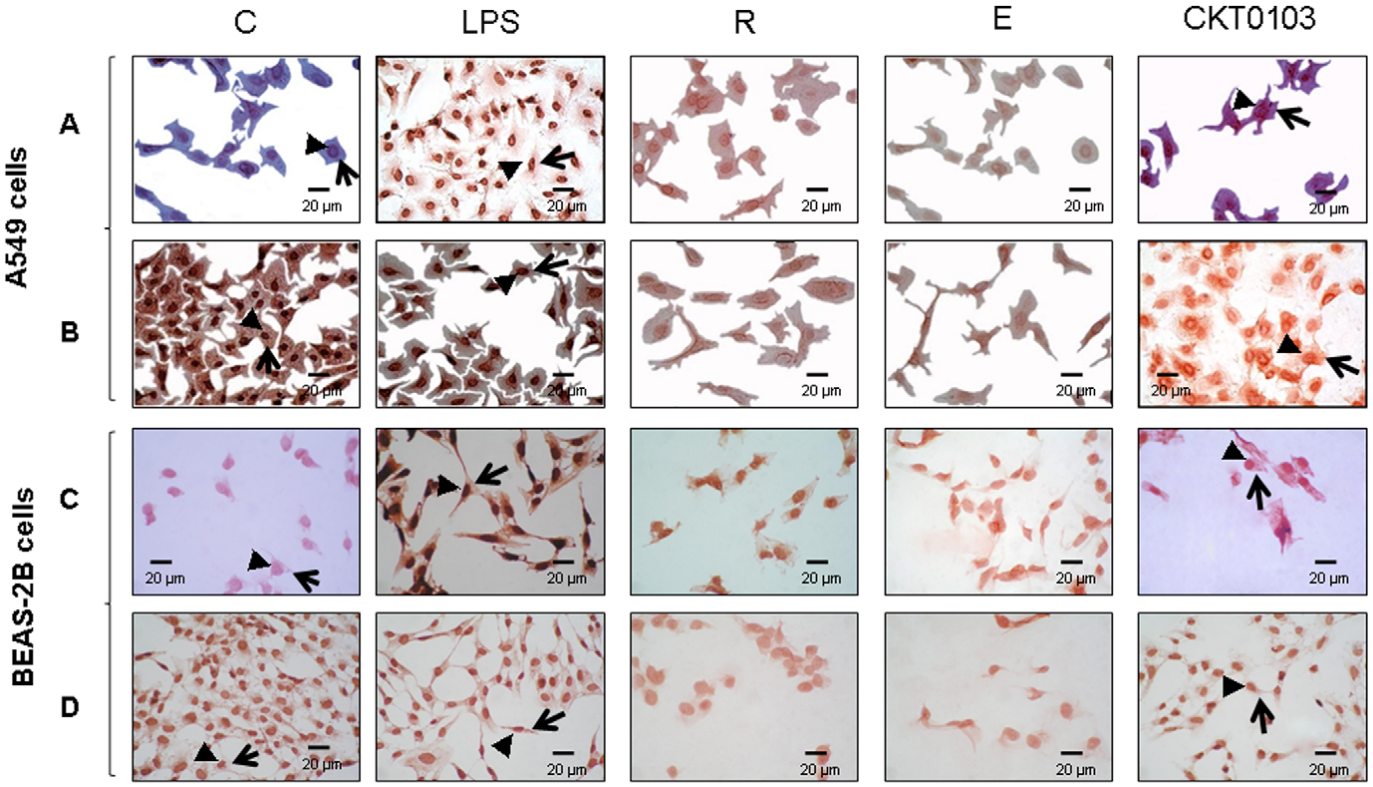
TLR4 (A,C) and Iκ B**α (B,D) protein immunocytochemical stainings in A549 (A, B) and BEAS-2B (C,D) cells stimulated with 100 ng/mL LPS (LPS) either in presence or absence of 10 µM rhein (R), emodín (E), CKT0103 (CKT0103) for 18 hours.** Red-pink color indicates positive staining (3-amino-9-ethylcarbazole) for TLR4 and IκBα proteins; blue/violet indicates nuclei counterstained with hematoxylin. TLR4 staining was found in nuclei (arrowheads) and cytoplasm (large arrows) in A549 and BEAS-2B cells treated with LPS but not in A549 and BEAS-2B control-vehicle cells or treated with LPS plus CKT0103. IκBα staining was found in nuclei (arrowheads) and cytoplasm (large arrows) in control-vehicle A549 and BEAS-2B cells and in A549 and BEAS-2B cells treated with LPS plus CKT0103. Strong immunostaining for TLR4 was observed in the LPS group and strong IκBα immunostaining was observed in control-vehicle A549 and BEAS-2B cells. Panels correspond to ×400 magnifications.

The expression of TLR4 was increased when A549 and BEAS-2B cells were exposed to LPS (p<0.001) (Figure 5). Co-treatment with rhein and emodin prevented the increase in TLR4 levels in both cell lines. CKT0103 significantly reduced TLR4 levels (p<0.001); this effect was significantly greater than either LPS+rhein (p<0.001) or LPS+emodin (p<0.001). In addition, exposure to LPS resulted in the degradation of IκBα in both cell lines (p<0.001); the decrease was markedly attenuated by CKT0103. Co-treatment with rhein or emodin did affect IκBα protein levels in a negative manner. However, CKT0103 produced a statistically significant increase of IκBα protein levels in A549 and BEAS-2B cells when compared to LPS (p<0.001 and p<0.05, respectively). Immunocytochemical staining for TLR4 and IκBα supported these findings (Figure 6).

## Discussion

A successful program aimed at discovering novel medicines requires reasonably reliable preclinical models. In anticancer drug chemotherapy, appropriate models have been developed [26], and this strategy has been applied for the past couple of decades by the NCI within the Development Therapeutics Program [11]. Based on this approach, we initiated a screening program to identify new candidate drugs directed at ALI/sepsis. In this study, we provide a reasonably rapid, simple, and reproducible method to screen novel anti-inflammatory compounds using an LPS-induced airway epithelium cell injury model. The main findings of this study are: (i) our screening approach was able to identify an interesting candidate; (ii) CKT0103, the candidate chosen inhibited LPS-induced effects in two *in vitro* models of endotoxin-induced airway epithelial cell injury; (iii) inhibition of cytokine secretion was associated with down-regulation of TLR4 and up-regulation of IκBα; and (iv) inhibition of LPS activity was achieved while cell viability and integrity was preserved during CKT0103 treatment.

We selected A549 and BEAS-2B cell lines as representative alveolar and bronchial epithelial cells; epithelial cells have been implicated in the pathogenesis of sepsis-induced ALI/ARDS [14]–[17], [19], [20], [23], [27]. In histological sections from patients with ARDS, one of the first lesions appears to be alveolar epithelial damage [28], and one of the most important mechanisms that determines the severity of lung injury is the magnitude of injury to the alveolar epithelial barrier [1]. Epithelial cells generate various immune effectors such as cytokines, chemokines, and antimicrobial peptides in response to inflammatory stimuli [23], [29] and airway epithelium controls lung inflammation and injury through the NF-κB [30]. A plethora of experimental reports have used human A549 alveolar and BEAS-2B bronchial epithelial cell lines to study the acute lung inflammatory response induced by LPS, as acceptable, validated and suitable *in vitro* airway epithelial injury models based on the first steps in the development of sepsis and ALI/ARDS [14]–[20], [23], [27]. We selected *E. coli* LPS treatment because it has been used in most endotoxin-induced lung injury model [7], [8], [31], [32] and LPS is a key pathogen recognition molecule for sepsis [7], [30] that induces apoptosis in lung cells [33]. Several previous reports evaluating the efficacy of compounds on the LPS-induced activation of proinflammatory cytokines in the lung have used a similar *in vitro* alveolar epithelial injury model with A549 cells [16] or prior to examining the *in vivo* anti-inflammatory effects [34].

Prior to screening of the compounds, we optimized our experimental conditions. For example, the amount of FBS in the culture medium had to be reduced to 2% in order to allow cell growth and to observe differences in effect between LPS-treated and untreated cells. To keep the method simple, test compounds were administered together with LPS and the exposure time set at 18 h. Two controls were defined: one for untreated cells incubated in the presence of vehicle-0.5% (v/v) DMSO (negative control), and one for cells exposed to LPS (positive control). The effect was defined as percent of survival (PS), where the negative control has a PS value of 100% and the positive control has a value of 0%. We also studied emodin and rhein as reference compounds. We selected these compounds since they are commercially available natural products present in traditional Chinese herbs, and have been shown to inhibit LPS-induced NF-κB activation and cytokine expression [35], [36]. They have been considered as potential candidates for the treatment of sepsis [22]. Based on this approach, we identified CKT0103 from a novel family of aryl carbamates and ureas, and further examined its impact using LPS-treated cells.

Sepsis and sepsis-induced ALI/ARDS are significant causes of morbidity and mortality worldwide. Over 40% of patients with sepsis go on to develop ALI/ARDS. Various strategies for suppressing the inflammatory response have been tested in clinical trials for the treatment of sepsis. However, these trials have thus far been largely unsuccessful [37], and identification of novel therapeutic approaches for sepsis and/or endotoxin-induced ALI is an area of intense investigation. Since sepsis-induced ALI/ARDS is both an infectious and an inflammatory process, studies have addressed therapeutic inhibition of inflammatory mediators [38], direct neutralization of LPS [39] and antimicrobial peptides [40]. There are a number of studies addressing the anti-inflammatory effects of new chemical compounds in the context of endotoxin-induced epithelial cell injury [41]–[44]; however, the mechanisms of action and indications are quite different than our compound.

TLR4, a member of the Toll-like receptor family, is expressed in airway epithelial cells [45] and has been shown to be the main upstream sensor for LPS *in vitro* and *in vivo*. Toll-like receptors play a central role in initiating the innate immune system and activating NF-κB [6]. Based on our previous animal studies [46], [47] showing a potential therapeutic role for signaling events related to the TLR4/NF-κB pathway, we pursued therapeutic targets induced by LPS-induced lung epithelial cell injury [32], [48]. Since previous *in vitro* studies using LPS-stimulated airway epithelial cells focused on activation of proinflammatory mediators and increased cytokine release [14]–[20], [39], we first examined the expression of proinflammatory cytokines IL-6 and IL-8. Elevated levels of these cytokines are found in patients with ARDS, and have been found to have a direct correlation with the severity of lung inflammation and mortality [49], [50]. We found that CKT0103 down-regulated the expression of IL-8 induced by LPS activation. While rhein and emodin interfered with the IL-6 overproduction induced by LPS, CKT0103 suppressed the effects of LPS. This is a relevant finding since lung levels of chemokines are increased in ALI/ARDS patients and in experimental models of acute lung injury [49], [51], [52] and levels of pulmonary pro-inflammatory cytokines correlate with mortality in experimental models of sepsis and in patients with sepsis-induced ALI/ARDS [49], [53]. Epithelial NF-κB activation is sufficient to promote airway inflammation during ALI/ARDS [54], [55]. NF-κB activation is mediated by IκBα degradation. We found that CKT0103 down-regulated TLR4, which inhibited down-regulation of IκBα induced by LPS. However, we cannot exclude the possibility that the activation of a TLR4-independent mechanism would induce pro-inflammatory cytokines.

In summary, we have described a fast, simple and reliable cell culture method to screen compounds as potential inhibitors of LPS-induced airway epithelial cell injury. A new family of LPS inhibitors was discovered from a subset of our library of natural and synthetic compounds. This approach identified a potential therapeutic role for CKT0103 which inhibited cytokine secretion as a result of down-regulation of TLR4 and up-regulation of IκBα in A549 alveolar cells and BEAS-2B bronchial cells. Although the urea derivative CKT0103 had a marked effect on LPS-induced inflammatory activity, the precise mechanism of action requires further studies. Such studies could include: LPS-binding studies, interference with several steps of the TLR4/NF-κB pathway, and an examination of their effects on *in vivo* model of sepsis-induced ALI/ARDS.

In conclusion, we have demonstrated that CKT0103, a novel compound from a family of aryl ureas, counteracted the pro-inflammatory activity of LPS by modulating the TLR4/NF-κB pathway in two *in vitro* LPS-induced airway epithelial cell injury models based on the first steps of the development of sepsis/ALI. These studies suggest that CKT0103 could be a potential therapy in the acute phase of sepsis and septic ALI/ARDS. These findings provide a basis for testing this novel compound in animal models of sepsis-induced acute lung injury.

## Supporting Information

Table S1 **Chemical structure of natural and synthetic compounds and percentage of survival (PS) of A549 and BEAS-2B cells stimulated with 100 ng/mL** ***E. coli*** **LPS in combination with: 1a–c: pyrimidinyl carbamates; 2a–h: pyrimidinyl ureas; R = chemical structure.**
(DOC)

Figure S1 **Survival curves of A549 and BEAS-2B cells after 6, 12 and 18 hours of 0.1-1.0-10-100 ng/mL** ***E. coli*** **LPS stimulation.** We used A549 and BEAS-2B cells as control-vehicle (C) cells incubated in the presence of vehicle-0.5% (v/v) DMSO. The values reported for *E. coli* LPS concentrations have been normalized to those for untreated (no LPS) control-vehicle cells. ***p<0.001 vs. control.
(TIF)

Figure S2 **Trypan blue exclusion assay.** The percentage of viable and non-viable cells in A549 and BEAS-2B cell populations after treatment with 100 ng/mL *E. coli* LPS in the presence or absence of different concentrations of CKT0103 (0-0.1-1-10-100-1000 µM) for 18 hours. ***p<0.001 vs. control (C); *†* p<0.001 vs. LPS.
(TIF)

File S1 (DOC)
