# Correction: Anti-Inflammatory Activity of a Novel Family of Aryl Ureas Compounds in an Endotoxin-Induced Airway Epithelial Cell Injury Model

**DOI:** 10.1371/annotation/c85ae0cb-3718-4a36-9d81-2f8e6a62b1e0

**Published:** 2012-11-13

**Authors:** Nuria E. Cabrera-Benitez, Eduardo Pérez-Roth, Milena Casula, Ángela Ramos-Nuez, Carla Ríos-Luci, Carlos Rodríguez-Gallego, Ithaisa Sologuren, Virginija Jakubkiene, Arthur S. Slutsky, José M. Padrón, Jesús Villar

There were errors in the Funding section. The correct funding information is as follows: This work was supported by grants from CIBER de Enfermedades Respiratorias, Instituto de Salud Carlos III, Spain (CB06/06/1088); Instituto de Salud Carlos III, Spain (PI 10/0393, PI 11/00840); Regional European Social Funds (FEDER). EPR holds a “Sara Borrel” contract from Instituto de Salud Carlos III, Spain. The funders had no role in study design, data collection and analysis, decision to publish, or preparation of the manuscript.

