# Supplementary material for: Anti-Inflammatory Activity of a Novel Family of Aryl Ureas Compounds in an Endotoxin-Induced Airway Epithelial Cell Injury Model
Source: PLoS One. 2012 Nov 8;7(11):e48468. doi: 10.1371/journal.pone.0048468 (PMC3493555; doi:10.1371/journal.pone.0048468)
Supplement: Table S1 — Chemical structure of natural and synthetic compounds and percentage of survival (PS) of A549 and BEAS-2B cells stimulated with 100 ng/mL E. coli LPS in combination with: 1a–c: pyrimidinyl carbamates; 2a–h: pyrimidinyl ureas; R = chemical structure. (DOC) [file pone.0048468.s001.doc]

**TABLE S1**

|  |  | **A549 CELLS** | **BEAS-2B CELLS** |
| --- | --- | --- | --- |
|  | Compound (10 µM) | PS (±SD) | PS (±SD) |
|  | **Rhein** | -55.3 (±13.50) | -54 (±14) |
|  | **Emodin** | 24 (±15) | 23.33 (±14.50) |
|  | **1a** R = OMe | 11.27 (±2.52) | 9.12 (±6.60) |
|  | **1b** R = OPh | 17.70 (±3.58) | 18.75 (±7.58) |
|  | **1c** R = OPh-*p-*Cl | 15.70 (±3.44) | 12.62 (±0.94) |
|  | **2a** R = NHPh | 16.75 (±8.27) | 14.50 (±3.71) |
|  | **2b** R = NHPh-*o-*Me | 11.75 (±4.34) | 8.5 (±5.06) |
|  | **2c** R = NHPh-*p-*OMe | 15.27 (±6.80) | 14.37 (±6.65) |
|  | **2d** R = NHPh-*p-*F | 12.20 (±3.82) | 11.25 (±10.21) |
|  | **2e** R = NHPh-*o*,*p-*diCl | 58.72 (±5.75) | 59.96 (±0.65) |
|  | **2f** R = NHPh-*m*-CF_3_ | 9.42 (±4.40) | 7.25 (±4.03) |
|  | **2g** R = NHPh-*o*-CO_2_H | 22.25 (±4.89) | 19 (±8.29) |
|  | **2h** R = | 19 (±8.98) | 1.87 (±12.19) |
