# Supplementary figures and images for: Anti-Inflammatory Activity of a Novel Family of Aryl Ureas Compounds in an Endotoxin-Induced Airway Epithelial Cell Injury Model
Source: PLoS One. 2012 Nov 8;7(11):e48468. doi: 10.1371/journal.pone.0048468 (PMC3493555; doi:10.1371/journal.pone.0048468)

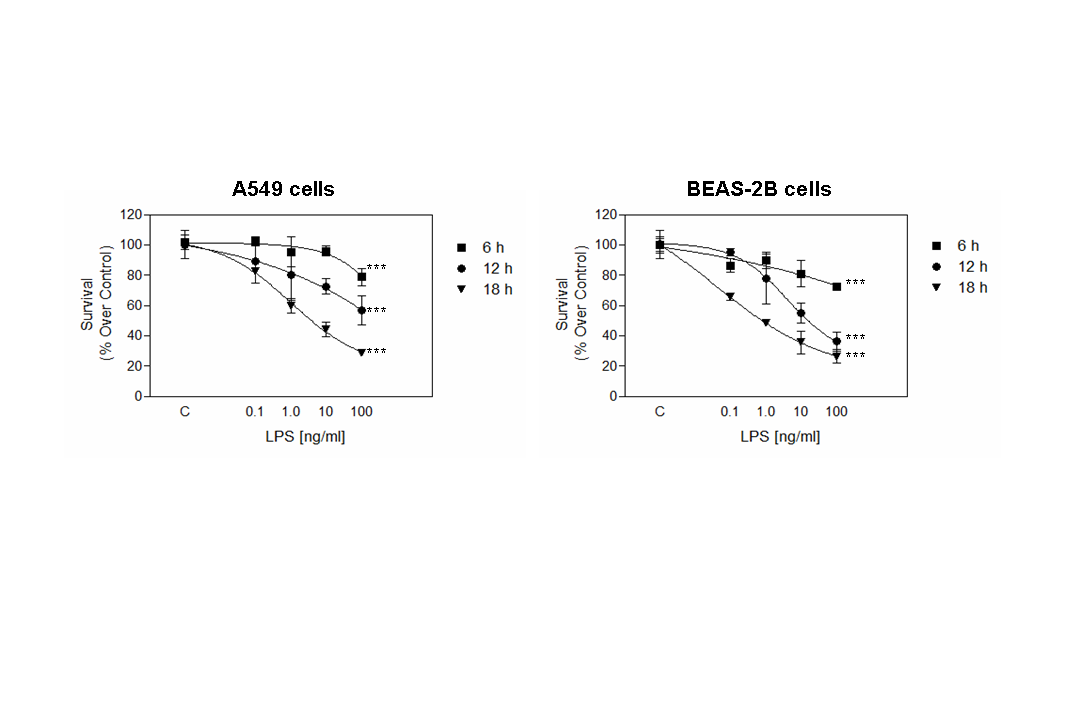

Supplement: Figure S1 — Survival curves of A549 and BEAS-2B cells after 6, 12 and 18 hours of 0.1-1.0-10-100 ng/mL E. coli LPS stimulation. We used A549 and BEAS-2B cells as control-vehicle (C) cells incubated in the presence of vehicle-0.5% (v/v) DMSO. The values reported for E. coli LPS concentrations have been normalized to those for untreated (no LPS) control-vehicle cells. ***p<0.001 vs. control. (TIF) [file pone.0048468.s002.tif]

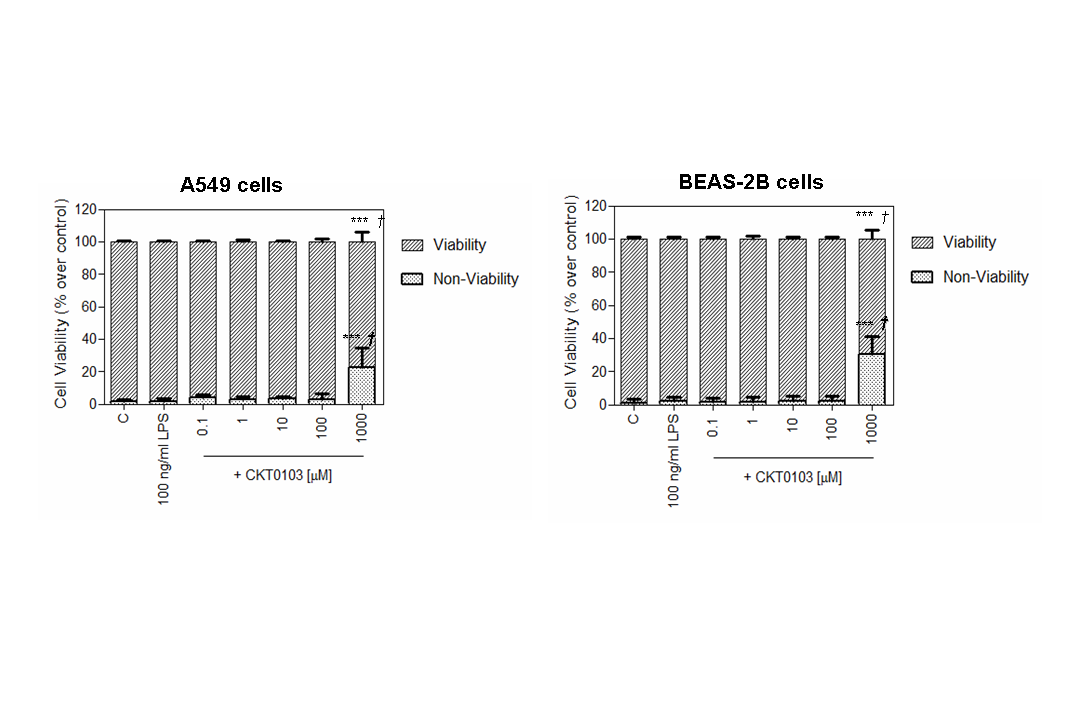

Supplement: Figure S2 — Trypan blue exclusion assay. The percentage of viable and non-viable cells in A549 and BEAS-2B cell populations after treatment with 100 ng/mL E. coli LPS in the presence or absence of different concentrations of CKT0103 (0-0.1-1-10-100-1000 µM) for 18 hours. ***p<0.001 vs. control (C); † p<0.001 vs. LPS. (TIF) [file pone.0048468.s003.tif]
