# Supplementary material for: Anti-Inflammatory Activity of a Novel Family of Aryl Ureas Compounds in an Endotoxin-Induced Airway Epithelial Cell Injury Model
Source: PLoS One. 2012 Nov 8;7(11):e48468. doi: 10.1371/journal.pone.0048468 (PMC3493555; doi:10.1371/journal.pone.0048468)
Supplement: File S1 — (DOC) [file pone.0048468.s004.doc]

***- ELECTRONIC SUPPLEMENTARY MATERIAL -***

**Anti-inflammatory activity of a novel family of aryl ureas compounds in an endotoxin-induced airway epithelial cell injury model**

**^1,2^ Nuria E. Cabrera-Benitez**, PhD

**^3^ Eduardo Pérez-Roth**, PhD

**^1,2^ Milena Casula**, PhD

**^1,2^ Ángela Ramos-Nuez**

**^3^ Carla Ríos-Luci**

**^4^ Carlos Rodríguez-Gallego**, PhD

**^4^ Ithaisa Sologuren**, PhD

**^5^ Virginija Jakubkiene**, PhD

**^6,7^Arthur S. Slutsky**, MD

**^3,*^ José M. Padrón**, PhD

**^1,2,6,*^ Jesús Villar**, MD, PhD, FCCM

From

*1) CIBER de Enfermedades Respiratorias, Instituto de Salud Carlos III, Madrid, Spain;*

*(2) Multidisciplinary Organ Dysfunction Evaluation Research Network, Research Unit, Hospital Universitario Dr. Negrin, Las Palmas de Gran Canaria, Spain;*

*(3) BioLab, Instituto Universitario de Bio-Orgánica “Antonio González” (IUBO-AG), Universidad de La Laguna, La Laguna, Spain;*

*(4) Department of Immunology, Hospital Universitario Dr. Negrin, Las Palmas de Gran Canaria, Spain;*

*(5) Department of Organic Chemistry, Faculty of Chemistry, Vilnius University, Vilnius, Lithuania;*

*(6) Keenan Research Center at the Li Ka Shing Knowledge Institute of St. Michael´s Hospital, Toronto, Canada.*

*(7) Interdepartmental Division of Critical Care Medicine, University of Toronto, Toronto, Canada*

**SUPPORTING MATERIAL AND METHODS**

**Reagents**

All chemicals used in this study were commercially available and research-grade. Dulbecco's modified Eagle's medium (DMEM) and RPMI 1640 medium were purchased from Flow Laboratories (Irvine, UK), Dulbecco’s modified Eagle’s medium (DMEN)/nutrient mixture F-12 Ham was from Sigma-Aldrich (Saint Louis, MO, USA), fetal bovine serum (FBS) was from Gibco (Grand Island, NY, USA), trichloroacetic acid (TCA) and glutamine were from Merck (Darmstadt, Germany), and Mueller Hinton broth was from Becton Dickinson (San José, CA, USA). Penicillin G, streptomycin, dimethylsulfoxide (DMSO), sulforhodamine B (SRB), MOPS, rhein, emodin, and *Escherichia coli* serotype 055:B5 were obtained from Sigma (St Louis, MO, USA). Protease Inhibitor Cocktail was purchased from Roche Molecular Biochemicals (Grenzacherstrasse, Switzerland), Bio-Rad DC Protein Assay from Bio-Rad Laboratories, CA and Coomassie staining from Invitrogen (Novex, Invitrogen, Carlsbad, California, USA). Restore Western Blot Stripping Buffer was obtained from Thermo Fisher Scientific Inc. (Rockford, IL, USA), Amersham ECL Western Blotting Detection Reagents from GE Healthcare, (Pittsburgh, PA, USA) and BD Cytometric Bead Array (CBA) Human Inflammatory Cytokines Kit from BD Biosciences (San Jose, CA, USA). AEC+ High Sensitivity Substrate Chromogen, Automation Hematoxylin Histological Staining Reagent were from Dako (Neue Flora, Hamburg, Germany) and HRP-Streptavidin Conjugate (Zymed, San Francisco, CA, USA).

Pure compounds were initially dissolved in DMSO at 400 times the desired final maximum test concentration of 100 µM. The final concentration of vehicle in culture medium did not exceed 0.5% (v/v). This concentration did not affect cell viability or cytokine production. In addition to the synthetic compounds, we used as reference drugs the natural products emodin and rhein. Both anthraquinone derivatives have shown inhibitory effects on LPS-induced inflammation in macrophages and chondrocytes [1].

**Cell lines, microbial strains and culture**

All synthetic carbamate and urea derivatives were screened against three widely used cell models: (i) a panel of six representative human solid tumor cell lines: A2780 [human ovarian cancer cell line, European Collection of Cell Cultures (ACACC, Salisbury, UK)], HBL-100 [human epithelial breast cells, Americam Type Culture Collection (ATCC), Manassas, VA, USA], HeLa [human cervical cancer cell line (ATCC, Manassas, VA, USA)], SW1573 [human lung epithelial alveolar cell carcinoma (ATCC, Manassas, VA, USA)], T-47D [human epithelial breast carcinoma (ATCC, Manassas, VA, USA)], and WiDr [human epithelial colorectal adenocarcinoma (ATCC, Manassas, VA, USA)]; (ii) a set of Gram-positive (*Staphylococcus aureus*, *Enterococcus faecalis*) and Gram-negative (*Escherichia. coli*, *Klebsiella pneumonia*) bacteria strains, and (iii) two fungal strains (Candida albicans, Candida glabrata).

A2780, HBL-100, HeLa, SW1573, T-47D, WiDr cell lines were a kind gift from Prof. Godefridus J. Peters (VU Medical Center, Amsterdam, Netherlands) and Dr. Rubén P. Machín (Hospital Universitario Dr. Negrín, Las Palmas, Spain). These cells were maintained in 25 cm^2^ culture flasks in RPMI 1640 supplemented with 5% FBS and 2 mM L-glutamine in a 37ºC, 5% CO_2_, 95% humidified air incubator. The bacterial and fungal strains were a gift from Dr. Julia Alcoba (Hospital Universitario NS Candelaria, Tenerife, Spain). Bacterial and fungal strains were grown on nutrient agar plates at 35‑37ºC.

A549 pulmonary alveolar cells [2] were a gift from Dr. I. Martinez-Gonzalez (National Microbiology Center, Madrid, Spain). Human lung bronchial epithelial cells (BEAS-2B) were purchased from American Type Culture Collection (ATCC, Manassas, VA, USA) and were maintained in Dulbecco’s modified Eagle’s medium (DMEN)/nutrient mixture F-12 Ham (DMEN/F-12) supplemented with 10% FBS and 2 mM L-glutamine in a 37ºC, 5%CO2, 95% humidified air incubator.

**Pilot studies**

Prior to the screening of the compounds, we performed pilot studies to set the in vitro LPS model and the experimental conditions. Thus, the amount of FBS in the culture medium had to be reduced to 2% in order to both allow cell growth and observe differences between LPS-treated and untreated cells. To keep the method simple, test drugs were administered together with LPS and the exposure time set at 18 h. Two controls were defined, one for untreated cells (negative control-vehicle) and one for cells exposed to LPS (positive control). The effect was defined as PS, where the negative control-vehicle has a PS value of 100% and the positive control has a value of 0%. We used emodin (1,3,8-trihydroxy-6-methylanthraquinone) and rhein (4,5-dihydroxyanthraquinone) as reference drugs. These two compounds are commercially available natural products that can be chemically classified as anthraquinone derivatives. These compounds were extracted from traditional Chinese herbs and have been considered potential candidates to treat sepsis [3]. For the purpose of this study, we decided to explore a small structure-focused library of aryl carbamates and ureas as potential drug candidates. The activity profile of the compounds did not show a clear structure-activity relationship. Compound CKT0103 was selected as drug lead for exploring in a more mechanistic manner the effects on LPS-treated cells. For comparison purposes, we also studied the activity profiles of emodin and rhein since several studies have shown their effects in the treatment of burn, infection, gallstone, hepatitis, inflammation, and osteomyelitis. Emodin was reported to inhibit LPS-induced NF-κB activation and inflammatory cytokine expression in RAW264.7 macrophages, and synergized with baicalin to suppress TLR4 and IL-6 expressions in pancreas and lung tissues of rats in the setting of acute pancreatitis [4,5]. Rhein was also found to exert anti-inflammatory roles by inhibiting IL-1β-induced activation of MEK/ ERK pathway and NF-κB and AP-1 in chondrocytes [6].

**Antiproliferative and antimicrobial assays**

Exponentially growing cells were trypsinized and re-suspended in antibiotic containing medium (100 units penicillin G and 0.1 mg of streptomycin per mL). Single cell suspensions displaying >97% viability by trypan blue dye exclusion were subsequently counted. Then, dilutions were made to give the appropriate cell densities for inoculation onto 96-well microtiter plates. Cells were inoculated in a volume of 100 µL per well at densities of 1 × 10^4^ (SW1573), 1.5 × 10^4^ (HeLa, A2780, HBL-100 and T-47D), and 2 × 10^4^ (WiDr) cells per well , based on their doubling times. Each agent was tested in triplicate at different dilutions in the range of 1–100 μM. The drug treatment was started on day 1 after plating. Drug incubation times were 48 h, after which time cells were precipitated with 25 μL ice-cold TCA (50% w/v) and fixed for 60 min at 4 ºC. Then, the sulforhodamine B (SRB) assay [7] was performed. The optical density (OD) of each well was measured at 492 nm, using BioTek’s PowerWave XS Absorbance Microplate Reader (VT, USA). Values were corrected for background OD from wells only containing medium. The percentage of growth (PG) was calculated with respect to untreated control-vehicle cells (*C*) at each of the drug concentration levels based on the difference in OD at the start (*T_0_*) and end of drug exposure (*T*), according to the National Cancer Institute (USA) formulas [8]. Therefore, if *T* is greater than or equal to *T_0_* the calculation is PG=100×[(*T*‑*T_0_*)/(*C*‑*T_0_*)]. If *T* is less than *T_0_* denoting cell killing the calculation is PG=100×[(*T*‑*T_0_*)/(*T_0_*)]. With these calculations three levels of effect could be determined; 50 % growth inhibition (GI_50_), total growth inhibition (TGI), and 50 % cell killing (LC_50_), that represent the concentration at which PG is +50, 0, and -50, respectively. Thus, a PG value of 0 corresponds to the amount of cells present at the start of drug exposure, while negative PG values denote net cell kill.

Bacterial and fungal strains were grown on nutrient agar plates at 35‑37ºC. After 24 h of incubation, cells were suspended in normal saline at a concentration of 5×10^5^ cfu/mL for bacteria and 1‑5×10^3^ cfu/mL for *Candida* spp. by matching with 0.5 McFarlands standards. Whole cell antimicrobial activity of compounds was determined in 96-well microtiter plates by a broth microdilution procedure using Mueller Hinton broth for bacteria and RPMI 1640 buffered with MOPS for fungi. Proper growth and sterile screening controls were included. Each compound was tested in duplicates at eight different 10-fold serial dilutions ranging from 100 to 0.05 μM. The microtiter plates were incubated at 35‑37 ºC in a moist dark chamber. After incubation, plates were shaken and the optical density values were recorded with a spectrophotometer (BioTek’s PowerWave XS Absorbance Microplate Reader, VT, USA). Bacterial plates were read at 630 nm after 24 h of incubation. Fungal plates were read at 490 nm after 48 h of incubation. The Minimum Inhibitory Concentration (MIC) was established as the concentration of compound that inhibited total growth when compared to untreated cells.

**Immunocytochemistry for TLR4 and IκBα**

A549 and BEAS-2B cells (9×10^5^ cells) were plated in duplicate chambers of an eight-chamber glass slide (Nunc® Lab-Tek® II Chamber Slide™) and cultured in the absence or presence of 100 ng/mL of LPS either alone or in combination with 10 µM rhein, emodin, and CKT0103 for 18 h. After incubation, cells were fixed in 4% paraformaldehyde in PBS for 20 min, washed twice with PBS and permeabilized with 0.2% Triton X-100 for 10 min. Primary antibodies directed against TLR4 and IκBα (Santa Cruz Biotechnology Inc, Santa Cruz, CA) were incubated at room temperature for 1 hour. Then, cells were washed in PBS and incubated for 12 minutes with a biotin-conjugated secondary antibody (Santa Cruz Biotechnology Inc, Santa Cruz, CA). Following another washing cycle, slides were incubated for 10 minutes at room temperature with streptavidin-horseradish peroxidase (HRP). Staining was visualized using the 3-amino-9-ethylcarbazole AEC+/substrate Chromogen. Finally, cells were rinsed in distilled water, counterstained with Mayer's hematoxylin, washed in running tap water. Coverslips with the cells were mounted on a slide with mounting media. Slides were viewed using an Olympus (BX50) microscope and photographed with an Olympus Camedia digital camera at x400 magnifications.

**LPS-induced airway epithelial cell injury model**

To study LPS-induced effects, exponentially growing A549 and BEAS-2B cells were inoculated in a volume of 100 µL per well at a density of 3×10^4^ cells per well. After 24 h, cells were exposed to E. *coli* LPS (0.1, 1.0, 10, 100 ng/mL) for 6, 12, 18 hours. Afterwards, cells were precipitated with 25 μL ice-cold TCA (50% w/v) and fixed for 60 min at 4ºC. Then, the sulforhodamine B (SRB) assay was performed as described previously. The optical density (OD) of each well was measured at 492 nm, using BioTek’s PowerWave XS Absorbance Microplate Reader. Data were expressed as 100% percentage over control-vehicle.

**A549 and BEAS-2B cells viability**

# The trypan blue exclusion method was performed to evaluate the viability of A549 and BEAS-2B cells in cell culture after 100 ng/mL E. coli LPS alone or in combination with a dose-curve of 0.1-1-10-100-1000 µM CKT0103. A549 and BEAS-2B cells were dispensed in a 25-mm culture plate at a density of 2×10^5^ per plate and in DMEN and DMEN/F-12, respectively supplemented with 2% FBS. After 24 hours incubation, cells were treated with 100 ng/mL *E. coli* LPS and with 0.1-1-10-100-1000 µM CKT0103 for 18 hours. A549 and BEAS-2B cells were diluted in trypan blue (TB, Sigma-Aldrich). TB negative (considered as viability cells) and TB positive (considered as non-viability cells) were immediately counted under light microscopy (Olympus CK-40 F-200) in triplicates using a hemocytometer (Neubauer counting Chamber. West Germany).

**Statistical analysis**

Statistical significance was assessed by one-way analysis of variance (ANOVA) followed by the Bonferroni post hoc test using SPSS (version 15.0 for Windows) when comparisons involving all experimental groups were performed. A probability of P<0.05 was adopted for statistical significance. Data are represented as mean±SD.

**SUPPORTING RESULTS**

**Antiproliferative and antimicrobial assays**

The results from antiproliferative activity toward human solid tumor cells, together with the antimicrobial test against bacteria and fungi strains revealed that all compounds were inactive (GI_50_ and MIC values >100 µM).

**LPS-induced effects on airway epithelial cells**

A549 and BEAS-2B cell survival decreased with increasing *E. coli* LPS concentration (Figure S1). The greatest decrease was observed in cells stimulated with 100 ng/mL *E. coli* LPS (p<0.001 for all time-periods).

**Effects of CKT0103 on A549 and BEAS-2B cells viability**

Only CKT0103 at a concentration of 1000 μM reduced A549 and BEAS-2B cells viability in a significant manner compared to the control-vehicle (p<0.001 in both cell lines) and compared to 100 ng/mL *E. coli* LPS (p<0.001 in both cell lines) (Figure S2).

**REFERENCES**

1. Liu X, Cheng J, Zheng X, Chen Y, Wu C, et al (2009) Targeting CpG DNA to screen and isolate anti-sepsis fraction and monomers from traditional Chinese herbs using affinity biosensor technology. Int Immunopharmacol 9: 1021-1031.

2. Lieber M, Smith B, Szakal A, Nelson-Rees W, Todaro G (1976) A continuous tumor-cell line from a human lung carcinoma with properties of type II alveolar epithelial cells. Int J Cancer 17: 62-70.

3. Karres I, Kremer JP, Dietl I, Steckholzer U, Jochum M, et al (1998) Chloroquine inhibits proinflammatory cytokine release into human whole blood. Am J Physiol 274: R1058-R1064.

4. Li HL, Chen HL, Li H, Zhang KL, Chen XY, et al (2005) Regulatory effects of emodin on NF-kappaB activation and inflammatory cytokine expression in RAW 264.7 macrophages. Int J Mol Med 16: 41-47.

5. Li Z, Xia X, Zhang S, Zhang A, Bo W, et al (2009) Up-regulation of Toll-like receptor 4 was suppressed by emodin and baicalin in the setting of acute pancreatitis. Biomed Pharmacother 63: 120-128.

6. Martin G, Bogdanowicz P, Domagala F, Ficheux H, Pujol JP (2003) Rhein inhibits interleukin-1 beta-induced activation of MEK/ERK pathway and DNA binding of NF-kappa B and AP-1 in chondrocytes cultured in hypoxia: a potential mechanism for its disease-modifying effect in osteoarthritis. Inflammation 27: 233-246.

7. Skehan P, Storeng R, Scudiero D, Monks A, McMahon J, et al (1990) New colorimetric cytotoxicity assay for anticancer-drug screening. J Natl Cancer Inst 82: 1107-1112.

8. Monks A, Scudiero D, Skehan P, Shoemaker R, Paull K, et al (1991) Feasibility of a high-flux anticancer drug screen using a diverse panel of cultured human tumor cell lines. J Natl Cancer Inst 83: 757-766.

**TABLE S1.**

|  |  | **A549 CELLS** | **BEAS-2B CELLS** |
| --- | --- | --- | --- |
|  | Compound (10 µM) | PS (±SD) | PS (±SD) |
|  | **Rhein** | -55.3 (±13.50) | -54 (±14) |
|  | **Emodin** | 24 (±15) | 23.33 (±14.50) |
|  | **1a** R = OMe | 11.27 (±2.52) | 9.12 (±6.60) |
|  | **1b** R = OPh | 17.70 (±3.58) | 18.75 (±7.58) |
|  | **1c** R = OPh-*p-*Cl | 15.70 (±3.44) | 12.62 (±0.94) |
|  | **2a** R = NHPh | 16.75 (±8.27) | 14.50 (±3.71) |
|  | **2b** R = NHPh-*o-*Me | 11.75 (±4.34) | 8.5 (±5.06) |
|  | **2c** R = NHPh-*p-*OMe | 15.27 (±6.80) | 14.37 (±6.65) |
|  | **2d** R = NHPh-*p-*F | 12.20 (±3.82) | 11.25 (±10.21) |
|  | **2e** R = NHPh-*o*,*p-*diCl | 58.72 (±5.75) | 59.96 (±0.65) |
|  | **2f** R = NHPh-*m*-CF_3_ | 9.42 (±4.40) | 7.25 (±4.03) |
|  | **2g** R = NHPh-*o*-CO_2_H | 22.25 (±4.89) | 19 (±8.29) |
|  | **2h** R = | 19 (±8.98) | 1.87 (±12.19) |
